# Neuroscientist’s Behavioral Toolbox for Studying Episodic-Like Memory

**DOI:** 10.1523/ENEURO.0073-24.2024

**Published:** 2024-08-27

**Authors:** Daniela Kunčická, Branislav Krajčovič, Aleš Stuchlík, Hana Brožka

**Affiliations:** ^1^Laboratory of Neurophysiology of Memory, Institute of Physiology, Czech Academy of Sciences, Prague 142 20, Czechia; ^2^Department of Physiology, Second Faculty of Medicine, Charles University, Prague 150 06, Czechia

**Keywords:** episodic memory, hippocampus, recollection, source memory, temporal binding, what–where–when

## Abstract

Episodic memory, the ability to recall specific events and experiences, is a cornerstone of human cognition with profound clinical implications. While animal studies have provided valuable insights into the neuronal underpinnings of episodic memory, research has largely relied on a limited subset of tasks that model only some aspects of episodic memory. In this narrative review, we provide an overview of rodent episodic-like memory tasks that expand the methodological repertoire and diversify the approaches used in episodic-like memory research. These tasks assess various aspects of human episodic memory, such as integrated *what–where–when* or *what–where memory*, source memory, free recall, temporal binding, and threshold retrieval dynamics. We review each task’s general principle and consider whether alternative non-episodic mechanisms can account for the observed behavior. While our list of tasks is not exhaustive, we hope it will guide researchers in selecting models that align with their specific research objectives, leading to novel advancements and a more comprehensive understanding of mechanisms underlying specific aspects of episodic memory.

## Significance Statement

Developing therapeutic approaches targeted at episodic memory loss, a debilitating condition significantly impacting daily life, requires a detailed understanding of episodic memory functioning. While advancements have been made on this front, conclusions often rely on a very limited set of tasks modeling only some aspects of episodic memory. This review addresses this gap by presenting an overview of 19 rodent behavioral tasks designed to assess key aspects of episodic memory testable in rodents. By offering researchers a broader behavioral toolkit adaptable to their specific research objectives, this review aims to facilitate novel insights and advancements in our understanding of episodic memory.

## Introduction

Episodic memory is a concept created to capture a phenomenon present in humans: the ability to recollect specific situations from the personal past (Tulving, 1983). If this faculty is compromised, as happens in Alzheimer’s disease or post-traumatic stress disorder, then the consequences are devastating. Choosing an appropriate animal model of episodic-like memory is essential to studying the relevant neural processes underlying episodic memory in detail. The most common species to study underlying mechanisms ofneuronal processes, including memory, are rodents. Rodents are indispensable in neuroscientific research for two main reasons. First, they are more readily available for experimentation. Second, numerous biotechnological tools are specifically applicable only to rodents. Consequently, high-throughput studies utilizing invasive, state-of-the-art biotechnologies for neuronal manipulation and visualization are feasible in rodents but significantly more challenging in other species. While contextual and trace fear conditioning tasks have gained prominence in this field (Liu et al., 2012; Jimenez et al., 2020; Trask et al., 2021; Bai et al., 2023), these tasks lack many key aspects of episodic memory, raising the possibility that insights gained may be specific to fear conditioning rather than episodic memory in general.

In this review, we aim to aid researchers in selecting rodent behavioral tasks that can provide insights into various aspects of human episodic memory. These aspects are testable characteristics of episodic memory regardless of whether animals possess human-like episodic memory. We first outline these aspects and subsequently establish criteria to rule out non-episodic mechanisms in the performance of episodic-like memory tasks. The second step is crucial, given the competing cognitive processes that can be harnessed to resolve the same behavioral challenges (Crystal, 2010). Next, we provide an overview of available tasks and critically appraise each from the standpoint of listed aspects and criteria. Some tasks meet more aspects and criteria than others, but insight might be gained from all sources—even when the task assesses just a single aspect of episodic memory. We conclude that no single task fully assesses episodic memory; instead, each task can be utilized to test one or several relevant aspects of episodic memory. However, many tasks are confounded by alternative explanations. Therefore, when appropriate, experimenters should exercise caution and employ suitable control groups and tests when using these tasks.

We note that a completely different but equally valid approach is to study animal episodic-like memory for its own sake. From this perspective, the term “proto-episodic memory” (Allen and Fortin, 2013) seems more fitting than “episodic-like memory” as the aim is not to model the human phenomenon. For proto-episodic memory, the picture might be quite different from human episodic memory—rats and other animals are not small humans, and concepts created to capture human phenomena might not fit best.

## Aspects of Episodic-Like Memory Suited for Neuroscience Research

When using a model of episodic-like memory, the initial consideration should center around which aspect of episodic memory the model aims to emulate. In the subsequent sections, we focus on the individual aspects of episodic memory that are well-suited for neuroscientific animal research. This discussion will exclude aspects that cannot be tested in animals, such as mental time travel (Zentall, 2006) and autonoetic consciousness (Dere et al., 2006), due to their inherent complexity and the limitations in assessing these phenomena in nonhuman subjects.

### Content of episodic memory: what–where–when information

Episodic memories involve remembering specific details of the event experienced, including what happened, where it happened, and when it happened (Tulving, 1972, 1985; Yonelinas, 2002). Therefore, knowing these three aspects of the previous personal experience indicates that the memory is most likely of episodic character. However, it is important to note that simply knowing the *what–where–when* of an event is neither a necessary nor sufficient indicator of episodic memory (Zentall et al., 2001). Memories meeting these criteria could still be semantic, such as historical facts that can often be described using the same criteria (Tulving, 1985; Zentall, 2013). Moreover, many human episodic memories may not include all three components of *what–where–when* content (Bauer et al., 2012; Allen and Fortin, 2013) as humans often show the absence of memory of when events occurred and their temporal order (Friedman, 1993, 2007; Friedman and Lyon, 2005; Eacott and Easton, 2010). Therefore, showing that animals remember the *what* and *where* of personal experiences may also sufficiently indicate episodic-like memory content.

As an alternative, Eacott and Easton (2010) suggested that the *when* component could be substituted by the context or occasion on which the event occurred—i.e., the *what–where–which* memory. In this sense, context refers to particular spatial, temporal, or perceptual details unimportant for the given episodic event (De Brigard et al., 2022). The advantage of demonstrating animals’ ability to remember the event’s context (*when* or *which*) lies in the fact that many events may have the same *what* and *where* components, but the context enables identifying the particular event (Clayton et al., 2003; Zentall, 2013).

In animal cognition research, a diverse array of *what–where–when* and *what–where–which* tasks have been developed to probe for episodic-like memory. These tasks capitalize on a range of behavioral paradigms, including “Foraging for food” section, “Mate-seeking” section, “Novelty recognition” section, “Contextual fear conditioning” section, or “Memory for the temporal order of events” section. While most of the remaining tasks we review provide evidence for *what–where* memory, they do not directly demonstrate that the animal remembers when the event occurred. However, they often offer advantages in simpler or quicker designs or the ability to simultaneously assess other aspects of episodic memory, such as “Temporal binding” section or “Incidental learning” section.

### Integrated memory content

Episodic memory should be a holistic representation in which all aspects of the memory are bound together and retrieved simultaneously (Clayton et al., 2003). This means that retrieving one aspect of the memory should also bring to mind other aspects of that memory (Metcalfe et al., 1992; Schwartz and Evans, 2001; Clayton et al., 2003). Several *what–where–when* and *what–where–which* tasks we review here are designed to test the binding of integrated memory representations (sections “Tasks assessing the integrated content of what–where–when memory” and “Temporal order of events”) another example being the task reported by Crystal and Smith (2014).

### Source memory (awareness of the learning context)

Source memory, closely linked with episodic memory, pertains to the origin of how memory was acquired (Johnson, 2005). It differentiates between memories generated internally, such as the intention to go jogging, and those of actual events. Source misattribution can significantly impact the accuracy of witness reports in criminal cases, as witnesses can incorporate details obtained later and misinterpret it as personal memory. Such erroneous attributions are known as confabulations (Johnson and Raye, 1998). Furthermore, source memory tends to deteriorate with age (Spencer and Raz, 1994; Cansino et al., 2019). Given the pivotal role of source memory in episodic memory, its understanding is crucial. Testing for the source tag is more straightforward in humans than animals; however, several such animal tasks were proposed, including tasks developed by Crystal et al. (2013) and Crystal and Alford (2014; section “Ruling out non-episodic memory mechanisms”).

### Independence from external cues (free recall)

Free recall assesses a person’s ability to retrieve memories without external cues (Tulving, 1985; Perner and Ruffman, 1995; Yonelinas, 2002). In a free recall task, human participants are typically presented with a list of items (e.g., words, pictures, or sounds); after a delay, they are asked to recall as many items as they can in any order. Evidence suggests that animals also have the capacity for behavior resembling free recall, as demonstrated by Menzel (1999) in his study on chimpanzees. In rodent models of episodic memory, free recall is not directly tested, but novelty recognition tasks can be viewed as testing similar theoretical constructs (section “Novelty recognition”). Nevertheless, the validity of the free recall paradigm is subject to debate, as individuals engaging in the free recall may still utilize internal cues despite the lack of overt external cues (McCabe et al., 2011; Unsworth et al., 2011). Furthermore, the majority of episodic recall is probably triggered by a specific combination of external cues, as demonstrated in studies on chimpanzees and orangutans (Martin-Ordas et al., 2013). Therefore, free recall may be an aspect intermittently present during memory retrieval but is by no means defining for episodic retrieval.

### Temporal binding capability

Episodic memories are often composed of sequences of events that do not occur simultaneously. The linking of temporally discontinuous events, temporal binding (Nombre and Coull, 2010; Sellami et al., 2017), is one of the hallmark features of episodic memory (Tulving, 1983, 2002; Kitamura et al., 2015; Eichenbaum, 2017; Ahmed et al., 2020). Although temporal binding is not always necessary when encoding episodic memory, as many memories of isolated events exist, understanding temporal binding enables us to understand the formation of complex episodic memories. Animal models that test temporal binding include, for example, order tasks (section “Temporal order of events”) and trace conditioning tasks (section “Trace conditioning”).

### Threshold retrieval dynamics

Episodic memory retrieval operates under threshold retrieval dynamics, as outlined by Eichenbaum et al. (2005) and Yonelinas et al. (2010). In this model, episodic memory recall is an all-or-nothing process: a subject does not remember anything about an experience until a certain threshold is reached; once this point is crossed, they recall the entire event, complete with its details. Conversely, familiarity-based recollection is gradual and graded, and the subject may not achieve full recognition. One way to distinguish between recollection and familiarity is the analysis of receiver-operating-characteristics (ROC) functions of recognition memory described by Yonelinas (2001). ROC analysis shows that humans use a mix of recognition and familiarity to solve item recognition tasks requiring them to differentiate between already seen and new items (Yonelinas, 2001). Fortin et al. (2004) designed an analogous task for rats in which they demonstrated that rats, similarly to humans, use both recognition and familiarity to solve the task (Eichenbaum et al., 2005). However, except for the task by Fortin et al. (2004; section “Threshold retrieval dynamics”), there is a scarcity of rodent tasks that directly assess threshold retrieval dynamics or utilize ROC analysis.

### Flexible planning for the future

It has been proposed that episodic memory primarily enhances future planning, as episodic memory lacks immediate adaptive value (Klein and Nichols, 2012; Szpunar et al., 2014). Future imagination and planning harness the same brain regions as episodic memory recall (Mullally and Maguire, 2014), and damage to episodic recall is almost always accompanied by deficits in future planning (Conway et al., 2016). When testing future planning in both humans and animals, care should be taken that the subjects plan for the future with a future motivational state in mind. According to Suddendorf et al. (2009), the greatest challenge of testing future thinking in animals is ensuring that an animal does not have a present desire for the reward and plans to obtain it for that reason.

As a skeptic, Suddendorf presented the Bischof–Kohler hypothesis, which posits that nonhuman subjects cannot imagine and act upon their future needs (Suddendorf and Corballis, 1997). Tulving (2005) introduced the concept of a “spoon test” to evaluate whether animals utilize the episodic memory system for future cognition. This test is inspired by an Estonian tale about a girl who, after failing to eat pudding in a dream due to the lack of a spoon, decides to place a spoon under her pillow in anticipation of a similar dream. According to Tulving (2005), an animal must demonstrate a similar foresight by preparing a tool for future use in a different location from where it was initially retrieved. The task must be, therefore, carried out with a specific future need in mind, independent of any current cues or needs. A number of studies have attempted to challenge the Bischof–Kohler hypothesis using the spoon test in several species, yielding convincing evidence of future planning in great apes (de Waal, 1982; Osvath and Osvath, 2008), corvids (Raby et al., 2007) and ravens (Kabadayi and Osvath, 2017), but not in rodents. The absence of valid future planning tasks in rodents could be explained by the requirement of extensive shaping in the tasks mentioned above (including tasks for apes and corvids). When shaping is required, it is difficult to exclude associative learning as a driver of observed behavior (Redshaw and Bulley, 2018). Moreover, impulsivity may overshadow future planning capacity in rodents, as it often overshadows this capacity in humans as well. As no convincing rodent future planning studies have been proposed so far, this review will not list any future planning tasks.

## Ruling Out Non-episodic Memory Mechanisms

Following the selection of a model, it is imperative to rule out explanations of the observed behavior that do not involve episodic mechanisms, as emphasized by Crystal (2010). This step is critical to ensure that the subsequent examination of neuronal substrates is specifically relevant to episodic memory. While other memory mechanisms may play a role, the task should not be solvable in the absence of an episodic memory mechanism (Crystal, 2021). Here, we have compiled a set of common issues researchers should consider to minimize the likelihood that the animal successfully solves a task using strategies and mechanisms irrelevant to episodic memory.

### Is the task hippocampus-dependent?

Our understanding of episodic memory circuitry has primarily been derived from human neurological case studies and, more recently, neuroimaging studies. The consensus is that the medial temporal lobe regions, including the hippocampal formation, perirhinal cortex, and parahippocampal cortex, are the crucial anatomical substrates for episodic memory function (Nyberg et al., 1996; Burgess et al., 2002; Kramer et al., 2007). While the involvement of the hippocampus is considered essential, it alone does not conclusively indicate the engagement of episodic-like memory processes (Eichenbaum et al., 2005). For instance, performance in the Morris water maze task, which is known to rely on an intact hippocampus (Morris et al., 1982), is not typically considered a measure of episodic-like memory. Therefore, a minimal prerequisite for proposing the involvement of episodic memory mechanisms is the observed dependency on or engagement of the hippocampus during task execution.

### Is the learning incidental? Can intentional learning during acquisition be excluded?

Incidental learning involves acquiring information unintentionally without realizing the significance of the information or intent to remember it. This process is characteristic of the formation of episodic memories (Zentall, 2013). To accurately assess incidental learning in humans, participants should not be pre-informed to memorize or expect a similar test (Kontaxopoulou et al., 2017), as this could lead to deliberate planning during a memory test, bypassing the incidental nature of learning (Zhou et al., 2012; Zentall, 2013). Martin-Ordas et al. (2014b) directly showed that both children and great apes performed better when they knew the information might be useful later, compared to situations where they learned it incidentally with no hint of future testing. In animal studies, it is crucial to avoid repeated testing of the same task, as this can cause animals to anticipate that the information might be required later. Such awareness shifts the learning process from incidental to intentional, which is carried out largely by distinct neurobiological mechanisms (Rugg et al., 1997; Kuhnert et al., 2013). In animal episodic memory research, incidental learning is specifically investigated in tasks where memory test occurs only once and the *unexpected question* tasks (section “Unexpected question tasks”).

### Can familiarity judgments be excluded as a means of solving the task?

Familiarity-based recall and episodic recollection-based recall are considered psychologically distinct (Mandler, 1980; Jacoby and Dallas, 1981) and involve different brain regions (Henson et al., 1999; Yonelinas, 2001 but see also Wang et al., 2018). In humans, the *remember/know* procedure is commonly used to distinguish between familiarity-based and recollection-based recall (Wixted, 2009). In animal research, it is important to evaluate each task to ensure that familiarity-based recollection cannot be utilized in favor of explicit remembering for task completion. There are several methods to eliminate the possibility that animals rely on familiarity rather than recollection in the given task. First, familiarity can be equated, rendering it ineffective for solving the task, as demonstrated in studies by Zhou and Crystal (2009) and Crystal et al. (2013). Alternatively, the task can be designed in a manner that effectively separates familiarity from recollection, as exemplified in research by Panoz-Brown et al. (2016, 2018). Next, the contributions of recollection and familiarity during a task recall can be determined by ROC analysis, which can be utilized in both humans and animals. Lastly, the evidence of disrupted learning following hippocampus lesions can effectively discount the familiarity explanation, as only episodic recall requires the intact hippocampus (Eldridge et al., 2000).

### Can short-term memory mechanisms be excluded?

For a task to qualify as an episodic-like memory task, it has been suggested that its acquisition should be separated from memory recall by at least 60 min to ensure that the task harnesses long-term memory (Pause et al., 2013). This period was considered essential for protein synthesis, which was believed necessary for long-term episodic memory formation. However, recent research has questioned the necessity of proteosynthesis for long-term memory formation (Hernandez and Abel, 2008; Ryan et al., 2015; Zhao et al., 2019), reducing the usefulness of the 60-min cutoff. Additionally, studies on short-term memory indicate that it typically operates within a timeframe of mere seconds to tens of seconds at most. Beyond this temporal threshold, long-term memory assumes control (Jonides et al., 2008). In summary, while there is no definitive rule, longer retention intervals are more conducive to engaging episodic memory mechanisms.

### Can conditioning be minimized?

In operant conditioning, subjects learn through reinforcing consequences to predict outcomes and optimize their actions to secure rewards or prevent punishments. A previously neutral stimulus becomes valuable in this process because it signals the likelihood of a reward or punishment. In certain tasks that aim to test episodic-like memory, it can be unclear whether the learned behavior results from episodic recollection or reinforcement learning. This ambiguity is particularly pronounced in tasks with repeated training trials. Strategies such as reducing the number of training trials or increasing the interval between the predictor stimulus and the reward or punishment are often employed to minimize the influence of reinforcement learning and to promote episodic-like processes. The discriminatory stimulus might also be internal, such as hunger or thirst if food or water restriction is used, respectively. The animals’ internal state can serve as a conditioned discriminatory stimulus the animal might use to “estimate the elapsed time” to solve the task, which might be wrongly interpreted as the *when* component of episodic-like memory. Nonetheless, reinforcement learning is not necessarily isolated from episodic memory. Research has indicated that a task may engage both episodic memory and operant conditioning, highlighting a complex interplay between these cognitive mechanisms (Dunsmoor and Kroes, 2019). In summary, when the task has few identical training trials and the interval between predictor and outcome is long, reinforcement learning and rule learning can be excluded as an alternate mechanism to episodic memory.

## Behavioral Tasks

In the following sections, we discuss specific rodent episodic-like memory tasks that can prove useful in probing neuronal substrates of episodic memory. For a behavioral task to be relevant in exploring the neural substrates of episodic memory, it should engage at least one aspect of episodic memory. The execution of this aspect should necessitate the utilization of episodic memory mechanisms, distinct from alternative mechanisms such as intentional encoding, reliance on relative memory strength (familiarity), short-term memory, or operant conditioning (section “Ruling out non-episodic memory mechanisms”). Furthermore, the experimenter should be aware that events to be remembered should occur only once; otherwise, processes essential in formation of “extended” or “general” events (Barsalou, 1988; Conway, 2001; Martin-Ordas et al., 2014a) will be involved. In the case of each task, we first review its essence and methodology, then discuss which aspects of the episodic memory the task tests and if it meets the exclusion criteria outlined above. To enhance clarity, we group and discuss tasks that share similar principles or utilize similar animal behaviors. If a task meets several categories, we place it into the most fitting category.

### Tasks assessing the integrated content of *what–where–when* memory

As we established in the previous sections, episodic memories contain details of the event experienced, including what happened, where it happened, and either when it happened or during which occasion it happened (event’s context). In this section, we review tasks that probe for the integrated content of *what–where*, *what–where–when*, and *what–where–which* memory utilizing a range of behavioral paradigms, including food-foraging, mate-seeking, novelty recognition, and contextual fear conditioning.

#### Food-foraging

##### Day, Langston, and Morris

**Figure 1.**
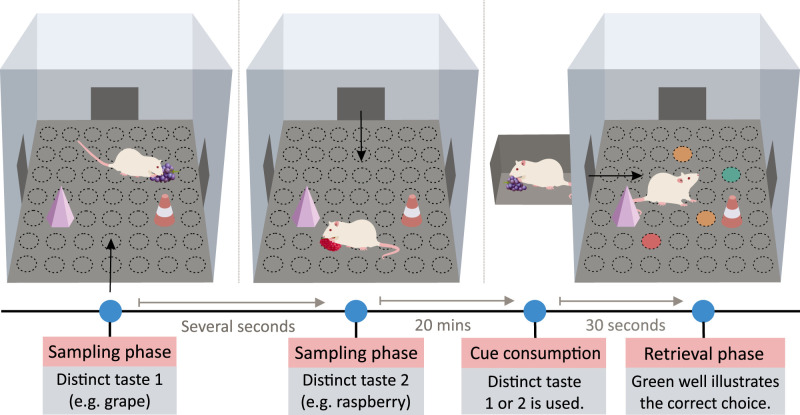
Overview of a food-locating task developed by Day et al. (2003). Rats navigated an event arena with visual cues they accessed from a different entrance during each visit (black arrows). In the sampling phase, rats learned the positions of two sand wells, each holding a distinct-tasting food pellet (e.g., grape and raspberry). Approximately 20 min later, rats entered the adjacent holding arena, consuming a cue pellet matching the taste of one of the previously eaten pellets (e.g., grape or raspberry). After that, rats explored the event arena with both previously open sand wells (green and red) and two new foil (orange) sand wells accessible. The task was completed if the rat dug in the sand well where it previously found the pellet matching the taste of the cue pellet (green).

Day et al. (2003) developed a rat task analogous to paired-associate tasks used to assess episodic recall in humans (Winocur and Weiskrantz, 1976; for an overview, see Fig. 1). In brief, rats consumed two food pellets of a distinct flavor, each located in a specific sand well of the event arena. Later, in an adjacent holding area, the rats were given a cue pellet of a particular flavor that matched one of the earlier pellets they consumed in the event arena. The rats then re-entered the event arena from a different direction and had to locate and dig in a sand well that had previously contained a food pellet matching the taste of the cue pellet. The authors showed that rats can accurately locate the correct sand well after a single taste-location pairing.

The task developed by Day et al. demonstrates the integration of memory for *what* and *where*. While the task itself can be conducted quickly, rats must undergo extensive behavioral shaping beforehand, which can take as long as eight months. Long and repeated training periods strongly suggest that the recall test is not unexpected, and learning of the taste-location association is not incidental. On the other hand, the task cannot be solved by familiarity as flavors and locations used on a given day were arranged equally familiar and counterbalanced in terms of order (i.e., which location and flavor from the pair was used first, second, etc., on a given day). The task likely harnesses long-term memory due to the delay between encoding and recall spanning at least 20 min. The performance impairment observed after hippocampal inactivation implies that the task relies on hippocampal-dependent mechanisms. In summary, the task designed by Day et al. is an original task that tests *what–where* memory and allows for experimental manipulations to assess animals’ performance before and after treatment; however, the time demands of the task have to be considered when designing further experiments.

##### Babb and Crystal

**Figure 2.**
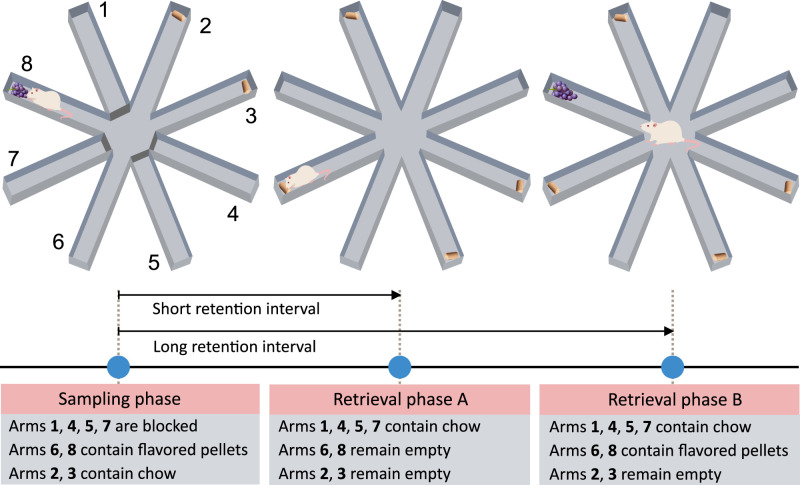
The overview of a food-locating task developed by Babb and Crystal (2006). The task assesses rats’ ability to remember the locations of distinctly flavored preferred food and predict its availability based on how long ago it was last consumed. In the initial phase, food pellets were placed at the end of four arms (2, 3, 6, and 8) while the remaining arms (1, 4, 5, and 7) were blocked. Two open arms contained chow pellets (2, 3), while the other two arms contained a food pellet of a distinct flavor (6—raspberry, 8—grape). Following a short delay (1 h), the previously blocked arms (1, 4, 5, and 7) opened and dispensed chow pellets, while the other arms remained empty. After a long delay (6 h), chow pellets in the previously blocked arms (1, 4, 5, and 7), as well as distinct-flavored pellets, were replenished (6, 8), meaning that distinctively flavored pellets were replenished after the long but not the short delay. During the task, food pellets were not visibly placed in the apparatus; rats were required to break a photobeam at each arm’s end to dispense the corresponding food pellet.

Babb and Crystal (2006) developed a food-locating task in which rats navigated a radial maze, where the location and flavor of food reward were linked to the duration of the retention interval (for an overview, see Fig. 2). In the task, distinctively flavored pellets (e.g., raspberry and grape) preferred by rats could be found in two specific arms and, upon consumption, replenished only after a long retention interval. Two other open arms contained chow pellets, and the four remaining arms were initially closed. Chow pellets were never replenished at their original locations; instead, they could be found after short and long retention intervals in the arms that were previously inaccessible.

The authors found that rats visit the locations of distinctively flavored pellets significantly more after the long than after the short retention interval. Moreover, they found that when one of the distinctive flavors is devalued by lithium chloride, or when rats were pre-satiated on the flavor, rats continue foraging for non-devalued distinctive flavored pellets but avoid the devalued ones. These findings imply that rats retain integrated *what–when–where* knowledge of past episodes and dynamically adjust their foraging strategies to bypass locations where they anticipate a lack of food or encounter undesirable food items. The task also utilizes long-term memory since rats had to retain knowledge of past experiences for several hours. However, since rats were subjected to numerous trials for each retention interval (approximately 80 training trials), the learning process was not incidental, and it cannot be excluded that rats were not aware of being tested on recall. In this regard, rats might have solved the task by employing simple rule learning: a short time has elapsed—search for the non-distinctive flavor; a long time has passed—search for the distinctive flavor. Moreover, rats could distinguish between an arm containing replenished food and an arm where food had not yet been replenished by utilizing memory trace strength to solve the task. Overall, the task designed by Babb and Crystal shows evidence that rats recall what, where, and when something happened, allowing them to flexibly apply past experiences to optimize their food-seeking behavior. However, it is important to consider the potential involvement of non-episodic mechanisms in this process.

##### Zhou and Crystal

Zhou and Crystal (2009) developed a compelling *what–where–when* episodic-like memory paradigm. During the study phase, which occurred either in the morning or afternoon, rats consumed a chocolate pellet at a distinct location within the 8-arm radial maze. After a variable delay, rats returned to the apparatus for the test phase. For one group of rats, the chocolate pellet was replenished if the study phase took place in the morning but not if it took place during the afternoon; these conditions were reversed in the second group. In subsequent probe experiments, Zhou and Crystal found that rats could remember and adapt their behavior based on the time of the day the study phase occurred, even when the delay between the study and test phase was extended from 2 min to 7 h, making the time of the test phase irrelevant to the reward’s replenishment. Moreover, Zhou and Crystal provided evidence that rats did not determine the time of the study phase based on the interval between light onset in the colony and the test session. This task, adeptly designed to assess *what–where–when* memory content, is particularly useful for investigating the mechanisms underlying the recall of the when component. However, it is crucial to recognize that even though the task is not exclusively solvable through familiarity judgments, the possibility that rats might utilize non-episodic mechanisms, such as intentional encoding influenced by conditioning in anticipation of the test session, cannot be excluded.

#### Mate-seeking

##### Ferkin and colleagues

Ferkin et al. (2008) developed a social task in which male meadow voles search for their mate based on their expectations of the mate’s current sexual receptivity. This task capitalizes on the fact that meadow voles are induced ovulators, where female meadow voles enter a short period of sexual receptivity shortly after giving birth (postpartum estrus; Clulow and Mallory, 1970; Meek and Lee, 1993). In this task, males were exposed to a two-chamber setup: one housing a pregnant female close to giving birth and the other a sexually unreceptive female (reference female). After 24 h, the males were reintroduced to the empty apparatus; at that point, the previously pregnant female would be in postpartum estrus (receptive). While exploring the apparatus, males strongly preferred the chamber of the female that would be in postpartum estrus. Another 24 h later, when the previously receptive female would be lactating (unreceptive), males showed a comparable preference for the lactating and reference female.

The fact that males showed a strong preference for the postpartum female but a similar preference for the reference and lactating female indicates that males remembered the *what–where–when* of previous events and flexibly used this information to estimate the current sexual receptivity of their mate. The rodent’s ability to locate the sexually receptive female shows the memory content was integrated and that they were aware of the learning context. The task meets several criteria of episodic memory. Learning occurred without repeated exposure to the event, reinforcement/rule learning could be excluded, and long-term memory was utilized. However, it is unknown whether males did not encode the location and sexual receptivity of both females intentionally, as this information holds significant ecological relevance driven by strong innate mating urges. The task developed by Ferkin et al. provide a simple yet valuable tool for assessing episodic-like memory in the social context, as it taps into animals’ ability to encode and retrieve ecologically valid contextual information over extended periods.

#### Novelty recognition

The novelty recognition tasks capitalize on rodents’ innate preference for exploring novel stimuli (Dellu et al., 1996; Bevins et al., 2002). When presented with a group of objects, rodents exhibit a preferential bias toward novel objects and less familiar objects (Silvers et al., 2007; Ennaceur, 2010)—the differential time spent exploring novel objects versus previously encountered objects is then used as a proxy for memory strength (Antunes and Biala, 2012). However, standard novelty recognition tasks are limited in providing definitive evidence of episodic-like memory, such as knowledge of the episode’s context (e.g., time and location). Moreover, novelty recognition tasks can generally be solved by relying on familiarity judgments or memory trace strength. To address these limitations, researchers have developed modified novelty object recognition tasks that retain the strengths of the original paradigm, including the absence of food deprivation or external motivation, while incorporating modifications that enhance their capacity to assess episodic-like memory processes.

##### Kart-Teke and colleagues

**Figure 3.**
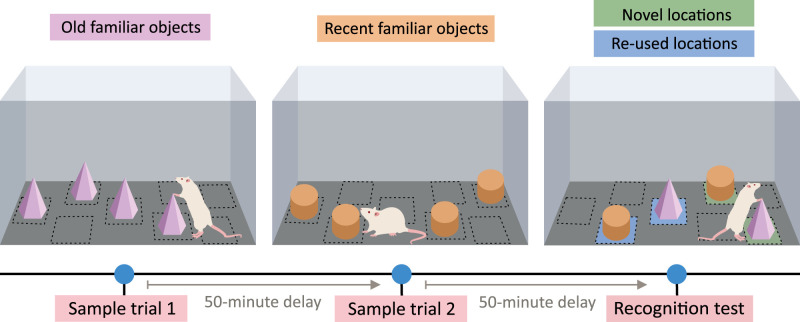
Overview of the three-trial object recognition task developed by Kart-Teke et al. (2006). The three-trial object recognition task assessed the ability of rodents to distinguish between objects based on where and how recently rodents encountered these objects. In the first sample trial, rats were presented with a set of four identical objects positioned at four out of eight possible locations within the apparatus. After 50 min, the rats re-entered the apparatus to encounter a new set of four identical objects: two placed at novel locations and two at locations occupied by the previous set of objects. Following another 50 min, the rats were reintroduced into the apparatus containing four objects: two of these objects were from the first set of objects and the other two from the second set; one object of each set was placed in its original location, while the other was relocated to a previously unused position. The time rats spent exploring each object during the test session was recorded and considered a measure of memory strength.

In the three-trial object recognition task by Kart-Teke et al. (2006), rodents distinguish between objects based on their degree of novelty (for an overview, see Fig. 3). In two sample trials separated by a 50-min delay, rodents encountered two distinct sets of four identical objects at specific locations within the arena. After another 50 min, rodents encountered these objects again during the recognition test: this time, they found two objects from each set of objects, with one object from each set occupying the same location as in the sample trial, while the other object from the set being placed at a novel location. This combination resulted in four objects differing in their degree of novelty to the rodent. The authors reported that rodents allocated most time exploring least recently encountered objects moved to novel locations, suggesting they distinguished between objects based on their memory of *what* (the object itself), *where* (the object’s location), and *when* (recency of encounter).

The rodents’ preference for less recently encountered objects over more recently encountered ones indicates temporal order memory, as described by Mitchell and Laiacona (1998). The task meets several other aspects of episodic-like memory as well. Rodents can recognize objects after extended delays, meaning the task utilizes long-term memory. Moreover, a single exposure to each object and its location during sample trials ensures that the learning is incidental and the recall test is unexpected. In a related study by Good et al. (2007b), object recognition based on familiarity was found to be independent of the hippocampus, while location-dependent object recognition was dependent on it. This implies that at least some aspects of the task performance likely involve the hippocampus, indicating processes relevant to episodic memory. However, the task can potentially be solved by relying on relative familiarity judgments. Each combination of attributes (how recently the object has been encountered and if it has been moved to a new location) results in a specific level of familiarity the animal can utilize to distinguish between objects. Overall, the three-trial object recognition task by Kart-Teke et al. provides a simple and efficient behavioral testing paradigm that taps into several aspects of episodic-like memory. Similar, but less complex, tasks were described by Dere et al. (2006) and Good et al. (2007a,b).

##### Eacott, Easton, and Zinkivskay

**Figure 4.**
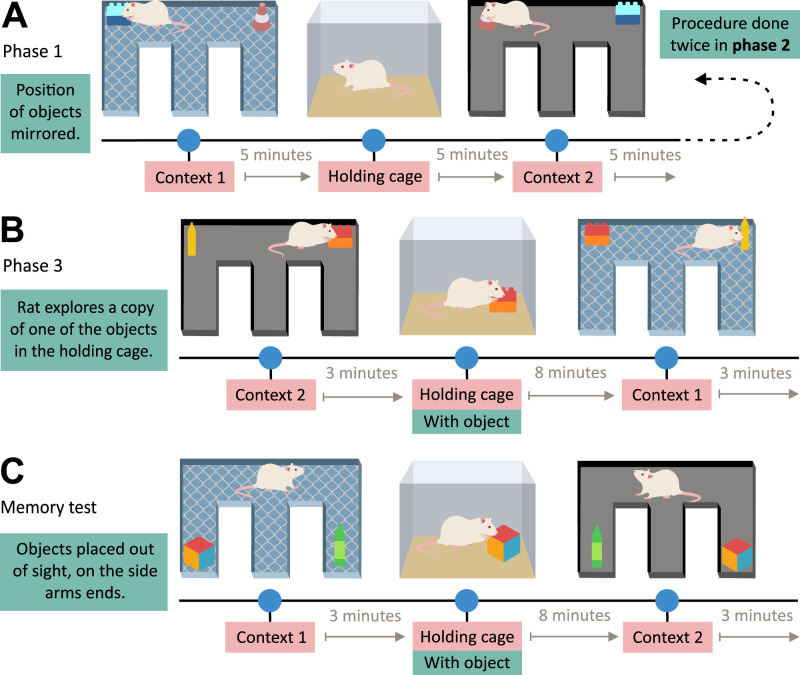
An overview of the task developed by Eacott et al. (2005). ***A***, In the first training phase, rats explored two distinct E-maze contexts, one smooth black and the other covered with wire mesh, each containing two visually distinct objects, and their location was mirrored across the two contexts. The exploration of two contexts was separated by several minutes, during which rats were placed in a separate holding cage. The second training phase (not shown) resembled the first, except that rats explored each context twice to learn that the location of objects remains stable given the same context even when visited repeatedly. ***B***, The third training phase was similar to the second, except rats were presented with a copy of one of the objects in the holding cage before being returned to one of the contexts. ***C***, The memory test occurred identically to the third training phase, but this time, both sample objects were placed at the ends of their respective side arms (behind the corner). The exploration of the (preferred) non-habituated object required rats to recall its location within the context, as it was not visible from the starting arm.

Eacott et al. (2005) investigated rats’ capacity to recall the locations of less explored objects in distinct environmental contexts. In principle, their task involved exposing rats to two distinct E-maze contexts, each containing the same pair of distinct objects, their locations mirrored across the two contexts and out of sight from the rats’ starting position (for an overview, see Fig. 4). Rats were then exposed to a copy of one of the objects in the adjacent holding cage for several minutes before being returned to one of the environmental contexts for recall. The authors reported that rats entered the arm containing the object that they did not just encounter in the home cage significantly above chance, indicating their memory for what (object), where (arm), and which (context). The ability of rats to navigate in the direction of the concealed preferred object implies that the memory of the object’s location was utilized flexibly depending on the recall context. In addressing the criteria to exclude non-episodic mechanisms, the concealment of objects negated the likelihood of rats relying on familiarity judgments. Moreover, above-chance success rates on the first attempt require the rats to remember the spatial location of the less familiar object within the maze context as neither object is visible from the starting point and their location is mirrored in each context. The incidental learning, marked by a solitary exploration in the home cage, suggests episodic memory engagement. Additionally, the task’s non-rewarded structure rules out reinforcement learning. Overall, the task by Eacott, Easton, and Zinkivskay is a robust method to assess episodic-like memory in rodents, as it effectively rules out non-episodic mechanisms and highlights incidental learning through its non-rewarded structure.

##### Leila Allen and colleagues

Allen et al. (2020) designed an incidental order task that leveraged the innate preference for less recently presented odors. In this task, wooden beads that were scented with odors from common kitchen spices were used as items to be remembered. Five scents were selected and presented on beads one by one in the home cage, with a 20-min delay between each item. After a 60-min delay period, a memory test was conducted, during which rats were presented with two scents from the sequence: the second versus the fourth. A preferential exploration of the bead carrying the scent presented earlier was considered an indication of sequential memory. Twenty minutes later, a classic novelty recognition test was conducted, this time comparing a known third scent from the original sequence against a new scent. A preference for the new scent indicated intact recognition of the previously presented scent. This single-trial task assesses the incidental encoding of odor sequences and taps into long-term memory. The authors demonstrated that performance in the sequential memory test decreased following lesions to the hippocampus, prefrontal cortex, and perirhinal cortex—regions important for episodic memory—while the performance in the classic novelty recognition test was spared. Moreover, the control memory trace strength test indicated the rats did not rely on memory trace strength in the sequential memory test; still, familiarity cannot be entirely ruled out. In summary, this task is an excellent, high-throughput method suitable to test incidental memory for the temporal order of events, relevant to episodic memory.

#### Contextual fear conditioning

Contextual fear conditioning is a type of associative learning in which a painful stimulus, usually an electric foot shock, is paired with a specific environmental context. Upon re-exposure, the paired context elicits a fear response, such as freezing behavior in rodents. Contextual fear memories can persist for months (Kim and Jung, 2006) and become generalized over time as the animal starts freezing in environmental contexts that resemble the original (Germer et al., 2019). This phenomenon of threat generalization mirrors patterns observed in human episodic memory (Starita et al., 2019). Similarly to episodic memories, contextual conditioning can be acquired incidentally and after a single experience. Despite these similarities, the original contextual fear conditioning paradigm fails to provide conclusive evidence of episodic-like memory, such as demonstrating knowledge of when the event occurred. Since contextual fear conditioning tasks assess non-specific behavior (freezing) as the main outcome and can be acquired in the absence of the hippocampus, the animals’ behavior can often be explained by non-episodic mechanisms such as familiarity or cue learning (Wiltgen et al., 2006; Baas et al., 2008). To overcome these limitations, researchers have proposed contextual fear conditioning tasks that incorporate modifications to more closely resemble episodic-like memory, such as awareness of the learning context and memory of when the aversive event occurred.

##### O’Brien and Sutherland

In a complex contextual fear conditioning task developed by O’Brien and Sutherland (2007), rats associated a foot shock with the context based on the time of the day they received the foot shock in that context. First, rats repeatedly explored two distinct contexts, one during the mornings and the other during the evenings. In the next phase, rats received a foot shock in a chimeric context consisting of parts of both contexts, A and B, either in the morning or evening. Then, at mid-day, rats were placed in one of the two contexts, and the duration of their freezing over the first several minutes was used to indicate the context-dependent retrieval of fear memories. O’Brien and Sutherland found that rats showed more fear in the context congruent with the time of day of the foot shock, suggesting that rats form an integrated memory of both context and the time of the day, with the memory of the context being flexibly updated by an aversive event that took place in that context. In this sense, the fear memory has *what–where–when* characteristics; the *what* component would correspond to foot shock, *where* to the context, and *when* to the time of the day. Moreover, the task is a one-trial learning event, and the learning of *what–when–where* information is incidental. Fulfilling the key criteria, this task is a valuable high-throughput task to be utilized in neuroscientific research investigating episodic memory in rodents.

##### Iordanova, Good, and Honey

Iordanova et al. (2008) utilized fear conditioning to investigate rats’ ability to form integrated memories. Rats were first exposed to two distinct environmental contexts, A and B, during both morning and afternoon sessions for 4 d. In the morning sessions, rats were presented with stimulus X (e.g., a tone) in context A and stimulus Y (e.g., a click) in context B. In the afternoon sessions, the presentation of stimuli was reversed, with stimulus Y presented in context A and stimulus X presented in context B. For the next 2 d at mid-day, rats were exposed to a novel context, C, where they received a foot shock following the presentation of stimulus X. On the final day, rats were assessed for contextual fear in contexts A and B during morning and afternoon sessions but without stimulus presentation. The authors found that rats showed increased freezing in either context but only when re-exposed to that context at the time of day when stimulus X was previously presented in that context (e.g., in the morning in context A and the afternoon in context B). These results indicate that rats formed integrated memories containing *what–where–when* elements (stimulus, context, time), acquired them through incidental learning, and with reliance on long-term memory. However, the task could also be solved through familiarity judgments, as it does not strictly require recollection. Moreover, each context exposure and stimuli pair presentation was conducted repeatedly. In summary, the study reveals rats’ capability to construct complex memories involving various elements, although familiarity as a means of solving the task is yet to be excluded.

### Tasks assessing temporal binding

The ability to link events in time is a crucial component of episodic memory. To assess temporal binding in animals, researchers have developed a variety of tasks that require animals to remember the order or temporal relationship between events. Such tasks are highly relevant to episodic-like memory because remembering the order of events preserves the “flow of events” as were experienced (Tulving, 1972, 2002). However, these tasks also have some drawbacks, such as requirements for extensive pre-training or shaping procedures.

#### Temporal order of events

##### Fortin, Agster, and Eichenbaum

Fortin et al. (2002) developed two tasks that assessed rodents’ ability to recognize novel odors and distinguish odors presented earlier in the sequential order. Both tasks began with a presentation of odors sequence, during which rats dug in five cups containing distinctly scented sand for a food reward. Each cup was presented individually to the rats in their home cages, with an inter-stimulus interval of 2.5 min. Following a brief retention interval of 3 min, rats were tested in the sequential order or recognition task. In the sequential order task, conducted first, rats were presented with two cups, each scented with a distinct odor presented earlier but occupying nonadjacent ordinal positions in the sequence. Rats were rewarded for digging in the cup scented with the odor that appeared earlier in the sequence. In the recognition task, conducted as the second, rats were presented with a cup scented with odor presented in the sequence and a cup with a novel odor; rats were rewarded for digging in the cup with the novel odor. The authors then tested the effect of hippocampus inactivation and found that it significantly impaired rats’ performance only in the sequential order task. While both tasks implemented a short retention interval, rats had to retain memory of the first odor in the sequence for at least 15 min, indicating the use of long-term memory. However, as both tasks required extensive behavioral shaping and repeated training trials, the rats could have memorized the sequence of odors intentionally. The design of the task also does not rule out solving the task using familiarity judgments or differences in memory trace strength. Overall, Fortin et al. provided compelling evidence that in their sequential order task, rats can memorize the temporal sequence of events and that this ability depends on the hippocampal activity, although non-episodic mechanisms cannot be definitively ruled out.

##### Ergorul and Eichenbaum

In the order task by Ergorul and Eichenbaum (2004), rats had to utilize the knowledge of the sequence of odors and their locations in the apparatus to receive a reward. During the sampling phase, a cup containing distinctly scented sand was placed in the apparatus at a randomly selected location. After the rats retrieved the reward from the cup, this process was repeated for three additional cups scented with different odors, each placed in a different location, and their presentation was separated by several seconds. After several seconds, two odors from the previously presented sequence were placed in the cups at their original locations on the platform. Rats had to dig in the cup scented with the odor they had encountered earlier in the sequence to receive the reward. Subsequently, probe trials were employed that exclusively relied on olfactory or spatial information: in the olfactory probe trials, only olfactory cues were accessible (the cups were placed adjacently at the center of the platform), whereas in the spatial probe trial, only spatial cues were available (the cups were filled with unscented sand but remained in their original locations).

The task provides compelling evidence of *what–where–when* memory: Rats’ ability to select the odor sampled earlier in the sequence suggests that rats could encode and retain the temporal order of events and, consequently, *when* each of these events occurred. Given that the rats’ initial choices (the cups they approached first) were correct more often than expected by chance, they could have relied on spatial cues to locate the target cup and then utilized odor cues or familiarity to verify their choice. The rats’ performance in the task was also adversely affected by the inactivation of the hippocampus, which led to statistically significant impairment in the classic version of the task, where both spatial and olfactory cues were crucial. During probe trials, however, hippocampal lesions only impaired spatial memory, not odor recognition, hinting toward limited episodic-like memory reliance.

Indeed, non-episodic strategies cannot be definitively ruled out. Besides distinguishing between locations based on relative familiarity (i.e. visiting the “less familiar” location each time), rats could utilize differences in memory strength to drive their behavior. In line with this explanation, rats undergo extensive training for the task, which could make them aware of the recall test and have them develop strategies to meet the task’s demands. The task also implements very short inter-stimulus and retention intervals (several seconds) which raises the possibility that rats could solve the task using short-term memory rather than episodic-like memory. In summary, Ergorul’s and Eichenbaum’s order task provides evidence of *what–where–when* memory in rats. However, further research, such as the implementation of longer retention intervals, is needed to rule out non-episodic mechanisms conclusively.

##### Timothy Allen and colleagues

Allen et al. (2014) devised an order task to investigate rats’ ability to identify odors presented out of sequence, specifically addressing the *what–when* aspects of memory. In their task, rats first gradually learned two sequences of distinct odors (e.g., ABCD and WXYZ) by nose-poking in an odor port. To indicate that an odor was in sequence, rats were trained to hold their nose in the odor port for 1 s, while to indicate an incorrect sequence, rats were taught to withdraw their nose immediately after odor presentation; correct responses were rewarded immediately after the rat’s response. For each animal, the stimuli A–D and W–Z always corresponded to the same stimuli (e.g., during all trials, the odor Y was “lemon,” odor B was “mint,” etc.). During the memory test, rats were presented with a sequence of odors and were required to determine whether each odor was presented in the *same sequence* as before (e.g., odor A is followed by odor B) or *out of sequence* (e.g., odor A is followed by odor C). Different types of *out of sequence* trials were used, such as repeats (e.g., ABAD), skips (e.g., ABD), or ordinal position transfers (e.g., AXCD) where one of the odors (X) originated from a different sequence (WXYZ) but occupied the same ordinal position.

In a parallel study in humans involving a sequence of images instead of odors, Allen et al. (2014) found that rats’ sequence memory performance closely mirrored that of humans, suggesting that the order task taps into aspects of memory shared by rodents and humans. Familiarity judgments or memory trace strength could be used to solve repeats (e.g., ABAD) and skips (e.g., ABD). Still, this approach would likely not be effective in detecting ordinal position transfers (e.g., AXCD, where X originally belongs to the WXYZ list). The mismatched odors would likely have the same or similar memory trace strength or be equally familiar because they occupy the same ordinal positions in their respective lists (e.g., B and X both occupy second positions). The differentiation between these odors also necessitate the animal’s awareness of the directional associative link with the preceding odor. However, the use of very short inter-stimulus intervals, typically in the order of milliseconds to seconds, raises concerns about the task’s reliance on episodic mechanisms. While the order task developed by Allen et al. shares several features indicative of episodic-like memory, the duration for which rats retain sequential memories and the rate at which their accuracy diminishes over time remain unclear.

##### Panoz-Brown and colleagues

**Figure 5.**
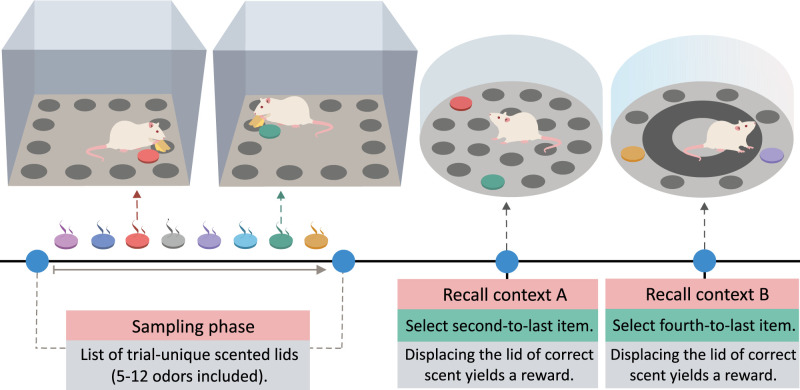
Simplified procedure of the odor order task by Panoz-Brown et al. (2018). The odor order task assesses the ability of rats to remember and retrieve the sequential order of odors in a context-dependent manner. During the sampling phase, rats were presented with a sequence of 5–12 unique odors within an event arena and without the knowledge of the definitive length of the sequence. After a 60-min retention interval, rats were placed in one of two distinct recall contexts (A and B) and presented with two odors previously encountered during the sampling phase. The correct odor selection (rewarded response) varied depending on the recall context: in context A, rats had to identify the second-to-last odor, while in context B, they had to select the fourth-to-last odor.

The order task developed by Panoz-Brown et al. (2018) assesses rats’ ability to recall specific ordinal positions of odor cues presented earlier (for an overview, see Fig. 5). In short, rats were presented with a sequence of odors of varying number of items. After an hour, rats had to choose between two odors in one of the two visually distinct environmental contexts. In one context, selecting the second-to-last odor from the sequence was rewarded; in the other, selecting the fourth-to-last odor from the sequence was rewarded.

Rats could identify the correct ordinal positions, suggesting they have formed and retained long-lasting memory for *what* and *when* and could flexibly utilize this memory to respond according to the rules of the particular retrieval context. The authors also showed that rats performed well even when atypical intervals between odors in the list were used, implying that rats did not use relative familiarity to solve the task. Furthermore, the unpredictable length of the odor lists and the context-dependent nature of the correct responses prevented the rats from predicting the ordinal positions during acquisition. This suggests that the rats relied on a more complex memory process, such as mental replay, to reconstruct the odor sequence and identify the correct ordinal positions. The authors demonstrated that hippocampal inactivation impaired performance in the task, further supporting the involvement of episodic-like memory mechanisms. In summary, the order task developed by Panoz-Brown et al. exhibit several hallmarks of episodic memory, including the ability to represent past events in a contextually specific manner, resistance to interference, and the capacity to retrieve the memory after a delay. These characteristics and the evidence that the hippocampus is crucial for successful task performance suggest that the order task could be a valuable tool for assessing mechanisms involved in episodic memory.

#### Trace conditioning

Trace fear conditioning tasks have helped us gain valuable insights into the neural underpinnings of episodic-like memory (Trask et al., 2021; Zhang et al., 2021; Bai et al., 2023). These tasks require animals to associate temporally separated events, testing mainly their temporal binding capacity (Misane et al., 2005; Sellami et al., 2017). Typically, animals are placed in a chamber and exposed to a conditioned stimulus (e.g., sound) followed by an empty interval (1–60 s), after which an unconditioned stimulus, usually an electric footshock, is administered. This procedure is repeated multiple times, with individual trials separated by several minutes. Trial-unique stimuli are typically not used—instead, the same conditioned stimulus is paired with the unconditioned stimulus across most, if not all, trials. After a delay (usually 24 h), the animal is placed in a different conditioning chamber and presented with the conditioned stimulus alone. Freezing behavior is measured and compared to a baseline to assess learning.

Trace fear conditioning displays some, but not all, aspects of episodic-like memory. Trace fear conditioning tasks show reliance on hippocampal function (Misane et al., 2005; Bangasser et al., 2006; Sellami et al., 2017) and utilization of long-term memory, but generally do not require conscious recollection of the experienced event. As traditional trace fear conditioning tasks measure freezing as the main outcome, the tasks can be generally solved using familiarity judgements. Moreover, repeated trials suggest intentional learning rather than incidental encoding. In summary, while trace fear conditioning is a valuable tool for studying temporal binding, it does not fulfill most of the criteria for testing the presence of episodic-like memory in animals.

##### Radostova and colleagues

**Figure 6.**
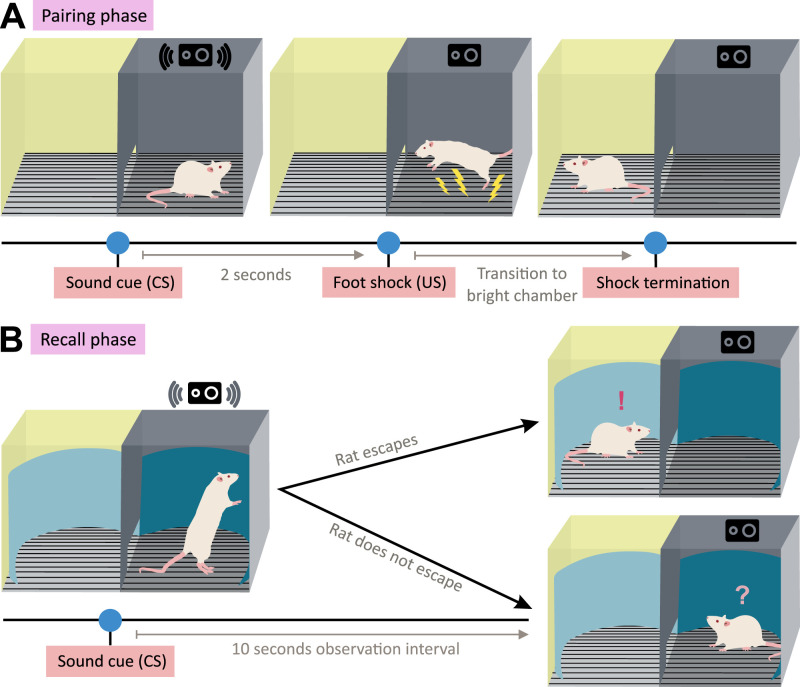
The procedure of the task developed by Radostova et al. (2023). During habituation (not shown), rats are familiarized with two visually distinct environmental contexts, A and B, each consisting of two interconnected chambers: a dark chamber and a brightly lit chamber. The chambers are separated by a partition that features a rectangular opening, enabling the rat to traverse between the two chambers. ***A***, In the pairing phase, which takes place in one randomly chosen context, a 2-s (80 dB) sound is played if at least 10 min elapsed and the rat is settled in the dark chamber. Two seconds after the sound ends, an electric foot shock (1 mA) is administered to the rat in the dark chamber, terminating upon the rat’s transition to the light chamber. ***B***, During recall, 24 h later, the rat is introduced to an environment distinct from that used during pairing. If at least 10 min elapsed and the rat settled in the dark chamber, then the 2-s sound cue was played again. The rat’s response to the sound is then observed—whether it escapes to the light chamber or remains in the dark chamber.

Radostova et al. (2023) developed a single-trial task that examines the ability of albino rats to associate a neutral and aversive stimulus separated by a temporal gap (for an overview, see Fig. 6). The task combines elements of one-trial learning, active avoidance, and trace conditioning and capitalizes on rats’ tendency to avoid an unpleasant stimulus (bright light) if there is no imminent threat (foot shock). First, rats were habituated to two distinct environmental contexts, each consisting of a dark and brightly lit chamber. Next, rats experienced a single pairing of a sound and a foot shock separated by 2 s while in the dark chamber of one of the environmental contexts. The next day, the rat’s reaction to sound was assessed while the rat resided in the dark chamber of the other environmental context (i.e., escaping from or remaining in the dark chamber).

The task exhibits several aspects of episodic-like memory. For instance, the task requires temporal binding due to the temporal gap between to-be-associated stimuli, and utilizes long-term memory as the pairing and recall phases were separated by 24 h. Rats’ escape to the brightly lit chamber upon hearing the sound cue during the test session suggests that they recalled the sound preceding the foot shock and, presumably, that the brightly lit chamber previously provided safety. Rats’ acquisition of this association and knowledge of the safety location could be considered incidental: rats were not pre-trained to respond to the sound in a specific manner and experienced the sound-shock pairing only once. Moreover, rats’ response to the sound cue by escaping to the brightly lit chamber in a different environmental context used during the test indicates the rat can utilize the acquired information flexibly. In conclusion, Radostova et al. introduced a conditioning task that requires temporal binding and presents the target stimulus once, ensuring that the learning is incidental.

### Source memory

Source memory, which refers to the recall of how memory was acquired—whether internally versus externally generated, or through observation versus experience—is a critical yet often overlooked aspect of episodic memory in rodent studies. Nonetheless, Crystal and colleagues have innovatively designed two experimental tasks to evaluate source memory in rodents. Both tasks require rodents to discern whether their location placement was self-initiated or if they were positioned by an experimenter. This distinction is key in assessing the integrity of source memory in these animals.

#### Crystal, Alford, and colleagues

In the task developed by Crystal et al. (2013), rats foraged for distinctive flavors of food that were replenished or not at their recently encountered locations according to a source information rule. In the first phase, rats were introduced to an eight-arm radial maze by being placed by an experimenter directly at a random food site, left to forage for food, and explore the maze for 10 min. Three arms of the maze dispensed regular chow, one dispensed highly preferred chocolate, and the remaining arms were closed. After a 4-min delay, rats were returned to the maze to forage for food again. Consuming chow required exploring previously closed arms while obtaining chocolate required remembering how it was acquired in the first phase. If the chocolate was self-obtained (the rat reached the chocolate site on its own), then it was replenished, but if the chocolate was obtained after the rat was placed directly at the chocolate site by an experimenter, it was not replenish. Rats revisited the replenishment location at a higher rate than the non-replenishment location while avoiding revisits to chow locations that never replenished, indicating that rats remembered *what* and *where* happened and the source of the encoded information. Subsequent experiments confirmed flexible memory usage (erasing room cues), long-term memory involvement (extending the delay to 7 d), and reliance on the hippocampal CA3 region. However, a non-episodic memory explanation exists for rats’ behavior, as rats could learn to prioritize encoding the replenishment location, avoiding the non-replenishment location as unnecessary for future visits (Crystal and Alford, 2014).

Building upon their previous work, Crystal and Alford (2014) modified this task to reduce potential bias toward encoding only specific information. Their first modification was adding a second chocolate site; at one of these sites, during the first phase, rats were placed directly by an experimenter, while at the other, the chocolate pellets were self-obtained. Another modification was the addition of a retrieval cue involving the presence or absence of chocolate pellets in the central hub at the start of the second phase. Which chocolate site was replenished depended on an interaction between first-phase chocolate pellet acquisition and the hub cue. For one group of rats, hub baiting with chocolate pellets meant that chocolate pellets from the self-obtained chocolate site would be replenished, while no pellets in the central hub indicated only the chocolate site to which an experimenter placed the rats was replenished; this contingency was reversed in the other group. In the subsequent experiment, the authors demonstrated rats form bound representations of what (flavor), where (location), source (self- or experimenter-generated), and context (room cues; Crystal and Smith, 2014). Despite increased complexity and training time, this setup demanded encoding of location, flavor, and pellet acquisition means for both chocolate sites, providing convincing evidence of source memory in rats.

### Threshold retrieval dynamics

The threshold retrieval dynamics characteristic of episodic memory posits that a certain recall threshold must be surpassed for successful recollection. While testing this concept in rodents presents challenges, one promising approach involves assessing rodents’ ability to retrieve memories under varying cue activation levels. The threshold for successful retrieval can be manipulated by adjusting factors such as the strength of the cue or the delay between encoding and retrieval. Subsequent ROC analysis can then be used to differentiate recollection from familiarity. Exploring this concept could provide valuable insights into retrieval failures when retrieval cues are present, as is the case in amnesia and dementia.

#### Fortin, Wright, and Eichenbaum

Fortin et al. (2004) designed a task for rats that evaluated the relative contributions of recollection—and familiarity-based retrieval mechanisms on odor recognition under varying response bias levels. The experiment presented rats with a list of distinct odors (presented one at a time) contained in identical open cups filled with scented sand. After 30 min, rats were individually presented in their home cages with cups scented with novel odors and odors from the previously sampled list in a randomized order. If the scent was novel, then rats received a reward for digging into the cup containing the novel odor; if the scent was from the list, then rats were rewarded for approaching an empty cup positioned at the back of their home cage. Throughout the sessions, the rats’ response criterion was biased by manipulating the test cup’s height and the reward amount provided for a correct choice. Subsequent ROC analysis revealed evidence of both recollection and familiarity contributing to rats’ odor recognition performance. After selective hippocampal damage, the rats’ performance declined, and their ROC curve analysis indicated an exclusive reliance on familiarity, providing support for the hippocampal involvement in odor recognition. The authors further demonstrated long-term memory involvement by extending the delay to 75 min; at this point, the ROC analysis revealed strong reliance on recollection over familiarity. However, as the task required extensive behavioral shaping and repeated training trials, rats likely expected the recall test, reducing the chance they learned the odors incidentally. Overall, the task by Fortin and colleagues provides a simple yet efficient tool for investigating the threshold retrieval dynamics of episodic memory.

### Unexpected question tasks

This section presents a set of behavioral tasks designed to examine animal’s memory by evaluating its ability to recall information unexpectedly and without explicitly indicating which information should be remembered. In most of these tasks, animals undergo initial training on two distinct tasks with shared characteristics. These tasks share a common limitation: the delay between the encoding of information and its subsequent retrieval is typically brief, preventing a definitive determination of the involvement of long-term memory mechanisms. The exception to this limitation is the task developed by Sheridan et al. (2024) who successfully combined unexpected question design with long retention intervals.

#### Zhou, Hohmann, and Crystal

**Figure 7.**
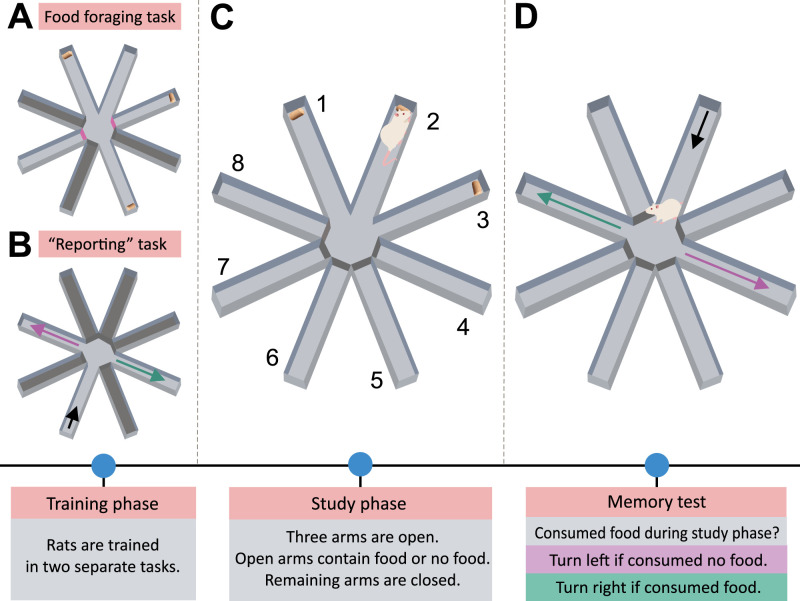
Overview of the task by Zhou et al. (2012). Rats were first trained on two distinct tasks. ***A***, In the food-foraging task, the rats learned to forage for food pellets by breaking light beams in a five-arm radial maze (light gray color). Initially, only three arms were accessible, with two additional arms opening after a retention interval (purple doors). Rats received a food pellet or no food pellet after breaking the light beam; the presence of food could not be determined until the sample arm was entered. ***B***, In the “reporting” task, rats learned to indicate whether they found food in the sample arm or not by entering the left or the right arm perpendicular to the sample arm (light gray arms). ***C***, In the probe trial, rats first performed the food-foraging task, searching arms 1, 2, and 3. ***D***, After visiting the three arms (either receiving food or not), two perpendicular arms opened (e.g., 4 and 8 if rat visited arm 2 as the last), offering the rat a choice between the left and right arm, similar to the “reporting” task.

Zhou et al. (2012) devised a task to assess rats’ ability to recall whether they had recently consumed food. They first trained rats on two distinct tasks using the same apparatus: (1) a foraging task involved foraging for food in a five-arm radial maze and (2) a T-maze-like task that required rats to “report” whether they received food in the sample arm by entering the left or right arm perpendicular to it (for an overview, see Fig. 7). In the probe trial, rats were first presented with the food-foraging task. Immediately afterwards, they were unexpectedly presented with the “reporting” task, requiring them to indicate whether they had consumed food during the preceding foraging task. The authors found that rats could accurately choose the corresponding arm, indicating that they could recall the presence or absence of food in the earlier task. As rats have never been explicitly tasked to “report” on the presence or absence of food in the radial maze task, encoding this information was likely incidental, and the subsequent memory test was unexpected. Despite the task not providing evidence of long-term memory recruitment, performance in the probe trials was impaired when the hippocampus CA3 region was inactivated. The radial maze task by Zhou et al. most likely requires processes relevant to episodic memory, as the dependence on the hippocampal activity and the unexpected nature of the memory test precludes the possibility that the animal uses non-episodic mechanisms to solve the task.

#### Nobuya Sato

**Figure 8.**
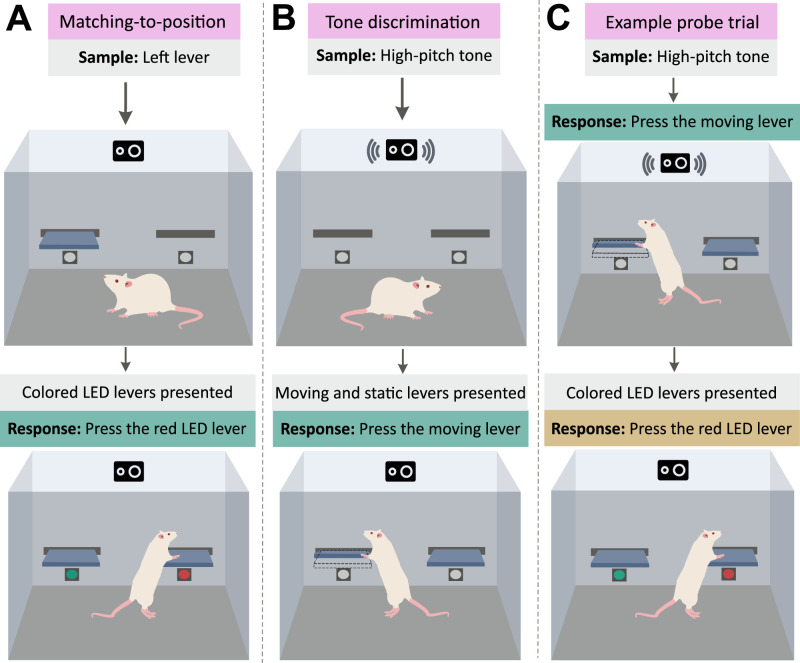
The example procedure of the tone-position task by Sato (2021). Rats were first trained in two distinct tasks. ***A***, In the delayed matching-to-position task, they learned to press a lever with a red or green LED tip that had been associated with the position of a sample lever presented earlier (left or right). For example, if the left lever was presented, the lever above the red diode was to be pressed, and vice versa. ***B***, In the tone discrimination task, rats learned to press a stationary or moving lever that had been associated with the pitch of a tone cue (high or low) presented 4 s earlier. For example, when a high-pitch tone was played, the moving lever was to be pressed, and vice versa. ***C***, Memory probe trials mimicked the tone discrimination task except no reward was provided for pressing the correct lever. Instead, two levers with illuminated LED tips were introduced into the apparatus, and the rat had to press the lever whose LED color matched the location of the lever pressed in the tone discrimination part of the trial. All combinations of the tone pitch, lever side, and LED color side were used, resulting in a total of eight probe trials.

The experiment reported by Sato (2021) combines two distinct behavioral tasks, utilizing a shared element to investigate rats’ ability to remember the location of their past actions. Rats were separately trained in a tone discrimination task, which required them to press a moving or static lever corresponding to the pitch of a presented tone, and a matching-to-position task, in which rats had to press a lever of color corresponding to the position of the sample lever presented earlier. During memory probe trials, rats performed a block of tone discrimination tasks before they were suddenly required to respond according to the rules of the matching-to-position task (for methodology, see Fig. 8). This memory test was likely unexpected since both tasks had never been combined before.

In the subsequent experiments, the author provided evidence that rats’ behavior was not driven by approach or avoidance responses (food was presented immediately after the first half of the trial) and that the rats’ performance requires an intact retrosplenial cortex. However, the four-second temporal gap between the rat’s lever-pressing behavior (in response to a tone cue) and the experimental question (the location of the pressed lever) falls within the rat’s working memory capacity, indicating that the task does not definitively demonstrate the involvement of long-term memory. It should be noted that another limitation of this paradigm is its labor-intensive nature, making it impractical for routine experiments (40 daily sessions, comprising up to 6,400 trials in each rat). In summary, Sato’s experiment effectively utilizes incidental learning and unexpected memory recall, but its laborious nature makes it challenging to implement in standard experimental settings.

#### Sheridan and colleagues

The task designed by Sheridan et al. (2024) further refines the design by Panoz-Brown et al. (2018; section “Temporal order of events”) by introducing an *unexpected question* dimension. Similar to the original task, rats were trained to identify the third-to-last odor in a variable-length sequence. Concurrently, they were trained in an eight-arm radial maze food-foraging task. The radial maze task consisted of a study phase where four baited arms were accessible, followed by a test phase where all arms were open, but food was only available in newly opened arms. During the probe trials, rats were presented with a radial maze with four baited arms, as in the study phase, but each bait was covered with a scented lid. Next, instead of the usual test phase, rats were placed in the environmental context used in the order task and were presented with two odors encountered during the study phase of the radial maze task. Rats were rewarded for correctly identifying the odor they visited as third-to-last in the radial maze task, as in the order task.

This design elegantly assesses a key aspect of episodic memory: the ability to retain and mentally replay sequences of temporally discontinuous events that were not intentionally encoded. Rats demonstrated remarkable flexibility, accurately answering the unexpected question after long retention intervals (15 min) despite its divergence from prior learning contexts. The high success rate observed in this task and its implication of mental replay for incidentally encoded information strongly suggest its utility in uncovering the mechanisms underlying episodic memory.

## Discussion

Episodic memory has multiple aspects including the characteristic content of what, where, and when something happened, incidental acquisition after a single experience, awareness of the learning context, independence of recall from external stimuli, threshold retrieval dynamics leading to sudden and comprehensive recall, and others. Our main aim was to provide an overview of rodent behavioral tasks that model these aspects of human episodic memory so that their neural underpinnings can be studied in detail. Many tasks we reviewed can be adapted to different experimental conditions or serve as an inspiration for developing tasks in other species, such as primates. At the same time, we aimed to stimulate researchers to think deeply and broadly when modeling the human phenomenon of episodic memory in animals.

As George Box famously noted, all models are wrong, but some are useful. An episodic-like memory model does not have to capture all aspects of episodic memory at once, or even the most crucial ones, to be relevant to the human phenomenon and help us understand the neural processes involved. For example, most tasks commonly considered “episodic-like” do not capture the basic fact that episodic memories are acquired incidentally during an episode that happened only once, but they require several training sessions. Despite this, the “non-incidental” episodic-like memory models are informative and shape our understanding of the neural processes involved in episodic memory. Moreover, even the study of processes prerequisite to, but not yet constituting, episodic memory can be relevant to understanding episodic memory.

Although a model does not need to be comprehensive, clarity is needed. Clarity about what selected aspects we aim to model and what was *actually* modeled—no single model currently available captures all aspects of episodic-like memory. Seeing the episodic-like memory research through the prism of various aspects, prerequisites, and graded relevance prevents undue generalizations and premature exclusion of new developments as irrelevant to episodic(-like) memory simply because a currently dominant aspect was not present or tested. Another venue is to focus on proto-episodic memory, i.e., evolutionary homologs of human episodic memory in other species for their own sake, not modeling the human phenomenon. We believe that a pluralistic approach to animal models, studying aspects besides the what–where–when memory, conceptual clarity, and frankness, are all crucial for advancing our understanding of episodic memory in all its complexity.

## Synthesis

Reviewing Editor: Mark Laubach, American University Decisions are customarily a result of the Reviewing Editor and the peer reviewers coming together and discussing their recommendations until a consensus is reached. When revisions are invited, a fact-based synthesis statement explaining their decision and outlining what is needed to prepare a revision will be listed below. The following reviewer(s) agreed to reveal their identity: Gema Martin-Ordas.

The review article was evaluated by 2 reviewers and myself as reviewing editor. We agree that the review is comprehensive with many strengths. The full reviews are appended below, to which I would request a point-by-point response to accompany your revised manuscript.

*Reviewer 1*

The manuscript provides a comprehensive overview of an important area of research. Key strengths include the comprehensive coverage, the weighing of strengths and weaknesses of approaches, and the assessment of utility of approaches for investigating rodent models of episodic memory. I support publication of the manuscript and offer some suggestions for the authors to consider while preparing a revision of the manuscript.

The manuscript ends with a brief discussion to note that no single approach addresses all issues that were considered in the review. Perhaps the authors could foreshadow this conclusion earlier in the manuscript.

Section 2. Can the authors outline a unified description of what is needed for episodic memory in an animal model? Currently, there are many elements described but the reader is not given guidance on which are essention, important, or extra details. Some authors have outlined a central hypothesis - for example Crystal 2021 writes that "the central hypothesis of an animal model of episodic memory is that, at the moment of the memory assessment, the animal remembers back in time to a specific earlier event or episode". The specification of a specific earlier event may help the reader evaluate the applicability of conditioning studies included in the review in Section 4.1.4 (some of which present the same event multiple times, making performance potentially based on processes that combine across repeated events rather than those that are about a specific earlier event).

Section 3. A key threat to an episodic memory proposal is that the animal may rely on "memory" rather than "episodic memory". Also from Crystal 2021 is: "we seek to compare the proposal that the animal is using a cluster of memory processes (e.g., working memory, semantic memory, associative memory, etc.) plus one other, namely episodic memory. This formulation needs to be compared to the same cluster of memory processes, with the notable absence of episodic memory. If the cluster that includes episodic memory -but not the smaller cluster- can explain the performance in a memory assessment, then the case for episodic memory is compelling." According to this perspective, there is nothing wrong with using some non-episodic memory processes as long as it can be shown that these alone are not adequate to explain performance.

Section 3.3 Another strategy is to equate familiarity. Examples include Zhou &Crystal 2009 and Crystal et al 2013. A further strategy involves dissociating familiarity and episodic memory. Examples include Panoz-Brown et al 2016 and 2018.

Section 3.4 Why is 60 minutes a criterion for long-term memory? Jonides et al 2008 and other citations would suggest a much shorter cutoff.

There are many places in the manuscript where the approach described does not adequately rule out memory strength or familiarity. Examples include sections that begin at Lines 312, 322, 344, 360, 432, 450, 473. Any case that involves comparing the age of memories likely has a memory trace strength or familiarity solution, unless some effort is taken to dissociate memory trace strength from episodic memory solutions.

Minor items:

L 96 - cite manuscript section 4.3 here

L 114-115 - the authors may be interested in a direct test of binding of episodic memory in Clayton, Yi &Dickinson 2001 and Crystal &Smith 2014.

L 256 - change "replenished" to "did not replenish"

L 371 - "potentially engages in temporal binding" - why? Omit.

L 420 - "without reinforcement" - why?

L 464-465 - is this statement about space, or odor, or both?

L 476 - state if ABCD was using trial-unique stimuli or always the same stimuli for a given subject. Same for WXYZ.

L 489 - familiarity is a potential solution. Any case that involves comparing the age of memories likely has a memory trace strength or familiarity solution, unless some effort is taken to dissociate memory trace strength from episodic memory solutions.

L 521 - note that trial-unique stimuli were not used.

L 533-552 - Radostova et al 2023 is a good example of a conditioning task because it presents the target stimulus once, rather than repeatedly. This should be noted here, and the presence or absence of this feature should be noted in other parts of 4.1.4

L 622-624 - Sheridan et al 2024 used a long retention interval.

L 670 - Note that Sheridan et al 2024 used a long retention interval.

References cited above that did not appear in the manuscript:

Clayton, N. S., Yu, K. S., &Dickinson, A. (2001). Scrub jays (Aphelocoma coerulescens) form integrated memories of the multiple features of caching episodes. Journal of Experimental Psychology: Animal Behavior Processes, 27(1), 17-29. https://doi.org/10.1037//0097-7403.27.1.17

Crystal, J. D. (2021). Evaluating evidence from animal models of episodic memory. Journal of Experimental Psychology: Animal Learning and Cognition, 47(3), 337-356. https://doi.org/10.1037/xan0000294

Jonides, J., Lewis, R. L., Nee, D. E., Lustig, C. A., Berman, M. G., &Moore, K. S. (2008). The Mind and Brain of Short-Term Memory. 59(Volume 59, 2008), 193-224. https://doi.org/https://doi.org/10.1146/annurev.psych.59.103006.093615

*Reviewer 2*

This is an excellent and very comprehensive review of the literature on episodic memory in rodents. My comments are very minor. PLease, see below:

Section 1 will greatly benefit if the authors clearly stated why rodents are good model for studying episodic memory.

Line 24. Please, cite Tulving here.

Lines 73-74. Can the authors indicate that the numbers in () refer to sections in the chapter?

Section 2.4. The authors mention that episodic memory should be independent from external cues. While this is a controversial area, I suggest that authors also include research on involuntary memory recall and discuss the work by Martin-Ordas et al. 2013 and other follow-up studies showing that great apes recalled past events when presented with particular combinations of external cues.

Lines 148-149. Could the authors speculate why they think there are no convincing tasks on future thinking in rodents, especially when these animals have been so successful in episodic memory tasks?

Lines 170-173. The authors might want to consider citing the work by Martin-Ordas and colleagues (2014) on prospective and retrospective memory-which gets at the issue of incidental learning in children and great apes.

Lines 178-187. The work by Hampton and colleagues on rhesus macaques should also be cited here.

Conclusion. The authors propose the tasks conducted with rodents as models to study episodic memory. Could the authors speculate about how adapting these tasks to other animal species might work? I am just thinking in terms of ecological validity, since some of the tasks presented here are designed taking advantage of rodents' behaviours?

## Author Response

Author's rebuttal Synthesis Statement for Author (Required):

The review article was evaluated by 2 reviewers and myself as reviewing editor. We agree that the review is comprehensive with many strengths. The full reviews are appended below, to which I would request a point-by-point response to accompany your revised manuscript.

Reviewer 1 The manuscript provides a comprehensive overview of an important area of research. Key strengths include the comprehensive coverage, the weighing of strengths and weaknesses of approaches, and the assessment of utility of approaches for investigating rodent models of episodic memory. I support publication of the manuscript and offer some suggestions for the authors to consider while preparing a revision of the manuscript.

1. The manuscript ends with a brief discussion to note that no single approach addresses all issues that were considered in the review. Perhaps the authors could foreshadow this conclusion earlier in the manuscript.

Response: Thank you for the suggestion. We added the following text to the Introduction section (Section 1, lines 46-49): "We conclude that no single task fully assesses episodic memory; instead, each task can be utilized to test one or several relevant aspects of episodic memory. However, many tasks are confounded by alternative explanations. Therefore, when appropriate, experimenters should exercise caution and employ suitable control groups and tests when using these tasks." 2. Section 2. Can the authors outline a unified description of what is needed for episodic memory in an animal model? Currently, there are many elements described but the reader is not given guidance on which are essential, important, or extra details. Some authors have outlined a central hypothesis - for example Crystal 2021 writes that "the central hypothesis of an animal model of episodic memory is that, at the moment of the memory assessment, the animal remembers back in time to a specific earlier event or episode". The specification of a specific earlier event may help the reader evaluate the applicability of conditioning studies included in the review in Section 4.1.4 (some of which present the same event multiple times, making performance potentially based on processes that combine across repeated events rather than those that are about a specific earlier event).

Response: Thank you for the suggestion. We outlined our 'central hypothesis' and added it to the preface of the Behavioral tasks section (Section 4, lines 248-254): "For a behavioral task to be relevant in exploring the neural substrates of episodic memory, it should engage at least one aspect of episodic memory. The execution of this aspect should necessitate the utilization of episodic memory mechanisms, distinct from alternative mechanisms such as intentional encoding, reliance on relative memory strength (familiarity), short-term memory, or operant conditioning (Section 3).

Furthermore, the experimenter should be aware that events to be remembered should 2 occur only once; otherwise, processes essential to form 'extended' or 'general' events (Barsalou et al., 1988; Conway et al., 2001; Martin-Ordas et al., 2014b) will be involved." 3. Section 3. A key threat to an episodic memory proposal is that the animal may rely on "memory" rather than "episodic memory." Also from Crystal 2021 is: "we seek to compare the proposal that the animal is using a cluster of memory processes (e.g., working memory, semantic memory, associative memory, etc.) plus one other, namely episodic memory. This formulation needs to be compared to the same cluster of memory processes, with the notable absence of episodic memory. If the cluster that includes episodic memory -but not the smaller cluster- can explain the performance in a memory assessment, then the case for episodic memory is compelling." According to this perspective, there is nothing wrong with using some non-episodic memory processes as long as it can be shown that these alone are not adequate to explain performance.

Response: We agree with this statement and highlighted it in the introduction to the exclusion criteria (Section 3, lines 172-174): "While other memory mechanisms may play a role, the task should not be solvable in the absence of an episodic memory mechanism (Crystal et al., 2021)." 4. Section 3.3. Another strategy is to equate familiarity. Examples include Zhou &Crystal 2009 and Crystal et al. 2013. A further strategy involves dissociating familiarity and episodic memory. Examples include Panoz-Brown et al. 2016 and 2018.

Response: Thank you for your suggestion. We added strategies to equate familiarity to the section about the exclusion of familiarity judgments (Section 3.3, lines 208-212): There are several methods to eliminate the possibility that animals rely on familiarity rather than recollection in the given task. First, familiarity can be equated, rendering it ineffective for solving the task, as demonstrated in studies by Zhou and Crystal (2009) and Crystal et al. (2013). Alternatively, the task can be designed in a manner that effectively separates familiarity from recollection, as exemplified in research by Panoz Brown et al. (2016, 2018).

5. Section 3.4. Why is 60 minutes a criterion for long-term memory? Jonides et al 2008 and other citations would suggest a much shorter cutoff.

Response: We included this information in the section about excluding short-term memory mechanisms (Section 3.4, lines 223-225): "Additionally, studies on short-term memory indicate that it typically operates within a timeframe of mere seconds to tens of seconds at most. Beyond this temporal threshold, long-term memory assumes control (Jonides et al., 2008)." 6. There are many places in the manuscript where the approach described does not adequately rule out memory strength or familiarity. Examples include sections that begin at Lines 312, 322, 344, 360, 432, 450, 473. Any case that involves comparing the age of memories likely has a memory trace strength or familiarity solution, unless some effort is taken to dissociate memory trace strength from episodic memory solutions.

3 Response: Thank you for the constructive feedback. We revised the listed tasks and checked whether each of them effectively discounts familiarity/memory trace strength solutions. Correspondingly, we included this information in their respective sections of the manuscript (as indicated by updated line numbers). • Introduction to novelty recognition tasks (lines 360-361): "Moreover, novelty recognition tasks can generally be solved by relying on familiarity judgments or memory trace strength." • Kart-Teke and colleagues (lines 384-386): "However, the task can potentially be solved by relying on familiarity judgments. Each combination of attributes (how recently the object has been encountered and if it has been moved to a new location) results in a specific level of familiarity the animal can utilize to distinguish between objects." • Eacott, Easton and Zinkivskay: The context-dependent (left or right) and concealed (not visible from the starting point) placement of objects likely negated the likelihood of rats relying on familiarity judgments (lines 402-404): "Moreover, above-chance success rates on the first attempt require the rats to remember the spatial location of the less familiar object within the maze context as neither object is visible from the starting point and their location is mirrored in each context." • Leila Allen and colleagues (lines 425-427): "Moreover, the control memory trace strength test indicated the rats did not rely on memory trace strength in the sequential memory test; still, familiarity cannot be entirely ruled out." • Fortin, Agster, and Eichenbaum (lines 502-503): "The design of the task also does not rule out solving the task using familiarity judgments or differences in memory trace strength." • Ergorul and Eichenbaum: Revising the task, we found some support for and some against non-episodic mechanisms involvement. We rewrote and expanded the text to reflect these findings (lines 522-535): "Given that the rats' initial choices (the cups they approached first) were correct more often than expected by chance, they could have relied on spatial cues to locate the target cup and then utilized odor cues or familiarity to verify their choice. The rats' performance in the task was also adversely affected by the inactivation of the hippocampus, which led to statistically significant impairment in the classic version of the task, where both spatial and olfactory cues were crucial. During probe trials, however, hippocampal lesions only impaired spatial memory, not odor recognition, hinting toward limited episodic-like memory reliance." "Indeed, non-episodic strategies cannot be definitively ruled out. Besides distinguishing between locations based on relative familiarity (i.e., visiting the 'less familiar' location each time), rats could utilize differences in memory strength to drive their behavior. In line with this explanation, rats undergo extensive training for the 4 task, which could make them aware of the recall test and have them develop strategies to meet the task's demands." • Timothy Allen and colleagues (lines 559-565): "Familiarity judgments or memory trace strength could be used to solve repeats (e.g., ABAD) and skips (e.g., ABD).

Still, this approach would likely not be effective in detecting ordinal position transfers (e.g., AXCD, where X originally belongs to the WXYZ list). The mismatched odors likely have the same or similar memory trace strength or are equally familiar because they occupy the same ordinal positions in their respective lists (e.g., B and X both occupy second positions). The differentiation between these odors also necessitate the animal's awareness of the directional associative link with the preceding odor." 7. Minor items:

1) L 96 - cite manuscript section 4.3 here.

Response: We added "(Section 4.3)" at the end of the sentence (line 107).

2) L 114-115 - the authors may be interested in a direct test of binding of episodic memory in Clayton, Yi &Dickinson 2001 and Crystal &Smith 2014.

Response: Thank you for your suggestion. We added a reference to the task by Crystal &Smith (2014) to the section about integrated memory content (Section 2.2, line 95) as another example of a task that tests the binding of an integrated memory representation.

While we did not create a separate subsection dedicated to this task, we previously referenced it as one of the two subsequent experiments conducted by Crystal and colleagues in Section 4.3. (line 667) that probed for source memory and memory content integration. We did not add a reference to Clayton, Yi &Dickinson 2001 as their task focuses on corvids.

3) L 256 - change "replenished" to "did not replenish" Response: We apologize for the confusion. The chow pellets were never replenished at their original locations (visited during the study phase); however, they became available after short and long retention intervals in the arms that were inaccessible during the study phase. We clarified the description of the task's method as follows (lines 294-297): "Two other open arms contained chow pellets, and the four remaining arms were initially closed. Chow pellets were never replenished at their original locations; instead, they could be found after short and long retention intervals in the arms that were previously inaccessible." 4) L 371 - "potentially engages in temporal binding" - why? Omit.

Response: We omitted this statement (line 422).

5 5) L 420 - "without reinforcement" - why? Response: We corrected the statement by omitting "without reinforcement" (line 473).

6) L 464-465 - is this statement about space, or odor, or both? Response: Thank you for your feedback. We clarified this statement as follows (lines 522-529): "The rats' performance in the task was also adversely affected by the inactivation of the hippocampus, which led to statistically significant impairment in the classic version of the task, where both spatial and olfactory cues were crucial. During probe trials, however, hippocampal lesions only impaired spatial memory, not odor recognition, hinting toward limited episodic-like memory reliance." 7) L 476 - state if ABCD was using trial-unique stimuli or always the same stimuli for a given subject. Same for WXYZ.

Response: The stimuli A-D and W-Z always corresponded to the same odors for a given subject. We clarified this information in the following sentence (lines 547-549): "For each animal, the stimuli A-D and W-Z always corresponded to the same stimuli (e.g., during all trials, the odor Y was 'lemon,' odor B was 'mint,' etc.)." 8) L 489 - familiarity is a potential solution. Any case that involves comparing the age of memories likely has a memory trace strength or familiarity solution, unless some effort is taken to dissociate memory trace strength from episodic memory solutions.

Response: We appreciate your comment. However, after carefully re-evaluating the experimental design, we conclude that familiarity alone would be sufficient to identify mismatches caused by skips and repeats but not ordinal transfer mismatches. We revised the text as follows (lines 559-565): "Familiarity judgments or memory trace strength could be used to solve repeats (e.g., ABAD) and skips (e.g., ABD). Still, this approach would likely not be effective in detecting ordinal position transfers (e.g., AXCD, where X originally belongs to the WXYZ list). The mismatched odors likely have the same or similar memory trace strength or be equally familiar because they occupy the same ordinal positions in their respective lists (e.g., B and X both occupy second positions). The differentiation between these odors also necessitate the animal's awareness of the directional associative link with the preceding odor." 9) L 521 - note that trial-unique stimuli were not used.

Response: Thank you for your suggestion. We added this information to the paragraph about the general design of trace conditioning experiments (lines 597-598): "Trial-unique stimuli are typically not used - instead, the same conditioned stimulus is paired with the unconditioned stimulus across most, if not all, trials." 6 10) L 533-552 - Radostova et al. 2023 is a good example of a conditioning task because it presents the target stimulus once, rather than repeatedly. This should be noted here, and the presence or absence of this feature should be noted in other parts of 4.1.4 Response: We highlighted your comment in a concluding sentence (lines 628-629): "Radostova et al. introduced a conditioning task that requires temporal binding and presents the target stimulus once, ensuring that the learning is incidental." We also revised the tasks in section 4.1.4 and included information about the absence of this feature in the task by Iordanova et al. (2008), which was previously missing (line 475): "Moreover, each context exposure and stimuli pair presentation was conducted repeatedly." 11) L 622-624 - Sheridan et al. 2024 used a long retention interval.

Response: To highlight that in contrast to other unexpected question tasks, the task developed by Sheridan et al. (2024) utilizes a long retention interval, we added the following sentence (lines 702-704): "The exception to this limitation is the task developed by Sheridan et al. (2024) who successfully combined unexpected question design with long retention intervals." 12) L 670 - Note that Sheridan et al 2024 used a long retention interval.

Response: Thank you for the correction. We revised the text as follows (lines 753-754): "Rats demonstrated remarkable flexibility, accurately answering the unexpected question after long retention intervals (15 minutes) despite its divergence from prior learning contexts." References cited above that did not appear in the manuscript: • Clayton, N. S., Yu, K. S., &Dickinson, A. (2001). Scrub jays (Aphelocoma coerulescens) form integrated memories of the multiple features of caching episodes.

Journal of Experimental Psychology: Animal Behavior Processes, 27(1), 17-29. https://doi.org/10.1037//0097-7403.27.1.17 • Crystal, J. D. (2021). Evaluating evidence from animal models of episodic memory.

Journal of Experimental Psychology: Animal Learning and Cognition, 47(3), 337-356. https://doi.org/10.1037/xan0000294 • Jonides, J., Lewis, R. L., Nee, D. E., Lustig, C. A., Berman, M. G., &Moore, K. S. (2008). The Mind and Brain of Short-Term Memory. 59(Volume 59, 2008), 193-224. https://doi.org/https://doi.org/10.1146/annurev.psych.59.103006.093615 7 Reviewer 2 This is an excellent and very comprehensive review of the literature on episodic memory in rodents. My comments are very minor. Please, see below:

1. Section 1 will greatly benefit if the authors clearly stated why rodents are a good model for studying episodic memory.

Response: Thank you. As you suggested, we added the following section to the introduction (Section 1, lines 27-32): "The most common species to study underlying mechanisms of neuronal processes, including memory, are rodents. Rodents are indispensable in neuroscientific research for two main reasons. Firstly, they are more readily available for experimentation. Secondly, numerous biotechnological tools are specifically applicable only to rodents. Consequently, high-throughput studies utilizing invasive, state-of-the-art biotechnologies for neuronal manipulation and visualization are feasible in rodents but significantly more challenging in other species. " 2. Line 24. Please, cite Tulving here.

Response: We added the missing citation (line 24).

3. Lines 73-74. Can the authors indicate that the numbers in () refer to sections in the chapter? Response: We added the word "Section" in brackets throughout the text in front of each section number to clearly indicate they refer to the sections of the manuscript.

4. Section 2.4. The authors mention that episodic memory should be independent from external cues. While this is a controversial area, I suggest that authors also include research on involuntary memory recall and discuss the work by Martin-Ordas et al. 2013 and other follow-up studies showing that great apes recalled past events when presented with particular combinations of external cues.

Response: Thank you for the suggestion. We added the following mention to the text concerning free recall (Section 2.4, lines 117-120): "Furthermore, the majority of episodic recall is probably triggered by a specific combination of external cues, as demonstrated in studies on chimpanzees and orangutans (Martin-Ordas et al., 2013).

Therefore, free recall may be an aspect intermittently present during memory retrieval but is by no means defining for episodic retrieval".

5. Lines 148-149. Could the authors speculate why they think there are no convincing tasks on future thinking in rodents, especially when these animals have been so successful in episodic memory tasks? Response: Thank you for the question. We elaborated the last paragraph of section 2.7 with the following (Section 2.7, lines 163-167): "The absence of valid future planning tasks in rodents could be explained by the requirement of extensive shaping in the tasks mentioned above (including tasks for apes and corvids). When shaping is required, it is impossible to exclude association learning as a driver of observed behavior (Redshaw 8 and Bulley, 2018). Moreover, impulsivity may overshadow future planning capacity in rodents, as it often overshadows this capacity in humans as well." 6. Lines 170-173. The authors might want to consider citing the work by Martin-Ordas and colleagues (2014) on prospective and retrospective memory-which gets at the issue of incidental learning in children and great apes.

Response: Thank you for the suggestion. We incorporated the citation into the text (Section 3.2, lines 194-196) as: "For example, Martin-Ordas and colleagues (2014a) directly showed that both children and great apes performed better when they knew the information might be useful later, compared to situations where they learned it incidentally with no hint of future testing." 7. Lines 178-187. The work by Hampton and colleagues on rhesus macaques should also be cited here.

Response: Thank you for the suggestion; however, we are unsure which citation you have in mind. For this reason, we did not include it in the text. However, we hope that it is not as seminal as to devalue the text in question.

8. Conclusion. The authors propose the tasks conducted with rodents as models to study episodic memory. Could the authors speculate about how adapting these tasks to other animal species might work? I am just thinking in terms of ecological validity, since some of the tasks presented here are designed taking advantage of rodents' behaviours? Response: Thank you for the suggestion. Many of the tasks are 'concepts' in the sense that they can be adapted to other species and experimental situations. We included the following remark in the concluding section (Section 5, lines 763-764): "Many tasks we reviewed can be adapted to different experimental conditions or serve as an inspiration for developing tasks in other species, such as primates."
